# Authorship and citation patterns of highly cited biomedical researchers: a cross-sectional study

**DOI:** 10.1186/s41073-023-00137-1

**Published:** 2023-09-05

**Authors:** Thomas Perneger

**Affiliations:** Division of clinical epidemiology, Geneva University Hospitals, Geneva, 1211 Switzerland

**Keywords:** Research assessment, Publications, Citations, H-index, Hm-index

## Abstract

**Background:**

Scientific productivity is often evaluated by means of cumulative citation metrics. Different metrics produce different incentives. The H-index assigns full credit from a citation to each coauthor, and thus may encourage multiple collaborations in mid-list author roles. In contrast, the Hm-index assigns only a fraction 1/k of citation credit to each of k coauthors of an article, and thus may encourage research done by smaller teams, and in first or last author roles. Whether H and Hm indices are influenced by different authorship patterns has not been examined.

**Methods:**

Using a publicly available Scopus database, I examined associations between the numbers of research articles published as single, first, mid-list, or last author between 1990 and 2019, and the H-index and the Hm-index, among 18,231 leading researchers in the health sciences.

**Results:**

Adjusting for career duration and other article types, the H-index was negatively associated with the number of single author articles (partial Pearson r -0.06) and first author articles (-0.08), but positively associated with the number of mid-list (0.64) and last author articles (0.21). In contrast, all associations were positive for the Hm-index (0.04 for single author articles, 0.18 for first author articles, 0.24 for mid-list articles, and 0.46 for last author articles).

**Conclusion:**

The H-index and the Hm-index do not reflect the same authorship patterns: the full-credit H-index is predominantly associated with mid-list authorship, whereas the partial-credit Hm-index is driven by more balanced publication patterns, and is most strongly associated with last-author articles. Since performance metrics may act as incentives, the selection of a citation metric should receive careful consideration.

**Supplementary Information:**

The online version contains supplementary material available at 10.1186/s41073-023-00137-1.

## Introduction

Citation-based indicators of research productivity are commonly used to evaluate scientists [1–3], and are often taken into account in funding, hiring and promotion decisions. Like any measurement system linked with reward mechanisms, these indicators may end up influencing the activity being evaluated, i.e., scientific research [4, 5]. Scientists who want to thrive, or merely remain active in their field, must be mindful of their indicators and must manage their activity so as to achieve acceptable targets [6]. This may have both desirable and unwanted consequences [6, 7].

Among the decisions scientists must make is how to allocate time and effort between their own projects, as principal investigators or members of a small team, or as more distant collaborators with others. In theory, an indicator that does not distinguish between levels of involvement will encourage activities that require the least effort per unit of measured output, be it an article or a citation, whereas an indicator that rewards in-depth scientific involvement may encourage researchers to invest their resources into fewer key projects. This implies that indicators should be chosen so as to assign value to the kind of scientific activity that policymakers wish to promote. The relationships between indicators and scientific activity have not received much attention to date.

The most common cumulative citation metric, the Hirsch index (H-index) assigns to all coauthors of any paper full credit from a citation, as though each co-author wrote the paper on their own [8]. This has been criticized by several experts as unfair [9–11]. One solution is to divide each citation credit among the authors of an article [12]. The Hm-index accounts for multiple authorship (hence the “m”) using this partial credit approach [13]. Hm starts with the same setup as H: articles are arranged in decreasing order of citations, but an article with k coauthors contributes only 1/k “article units” to the ongoing count. E.g., if a researcher has written 5 articles that have received 10, 5, 3, 2 and 1 citation, their H is 3. If these articles were written by 1, 2, 3, 4, and 5 authors (in the same sequence), Hm would allocate 1, 0.5, 0.33, 0.25 and 0.2 article units to each co-author, and the cumulative total for the index researcher would be 1, 1.5, 1.83, 2.06 and 2.26. The highest total that is less than or equal to the number of citations is 1.83, thus the researcher’s Hm would equal 1.83.

It is currently unclear whether cumulative citation metrics such as H or Hm reflect differences in authorship patterns, and if so what authorship patterns produce the highest values of citation metrics. At stake is the fairness and integrity of the research evaluation system. Since both H and Hm aim to measure research impact, presumably both metrics should rank researchers in a similar way. If that were not the case, this would mean that each metric reflects a different type of impact; understanding this difference is essential if the metrics are used for real-life decisions. At a minimum the relationships between research outputs and each citation metric should be acknowledged, and made known both to evaluation bodies and to those being evaluated, in order to ensure a fair interpretation of the results [14]. Furthermore, evaluation bodies may opt for the citation metric that best reflects the type of research activity they wish to promote.

The H and Hm indices also differ in their vulnerability to manipulation. The H-index puts no limit on the number of co-authors who will receive credit from a citation, and thus provides no disincentive to adding co-authors who may not have contributed substantially to the work. In contrast, the Hm-index splits a single article unit among all co-authors, so that adding a co-author who may not have contributed substantially comes at a cost to those who have. This property of the Hm-index may discourage undeserved authorship. The downside is that usage of the Hm-index may lead to excluding from the byline contributors who should be authors, per authorship guidelines [15].

In this study I examined the associations between the publication records of leading researchers in the health sciences – i.e., the numbers of articles written as single author, first author, mid-list author, or last author – and their H-index and Hm-index. Based on the incentives inherent in each indicator, I expected highest values of H to be associated with the largest total number of articles, and with large numbers of mid-list authorships; conversely, I expected the highest values of Hm to be associated with larger numbers of first and last author positions.

## Methods

### Design and sample

This cross-sectional study is a secondary analysis of the comprehensive database of researchers made available by Ioannidis et al. [16]. The database contains records of 159,683 researchers of all disciplines, identified through their publications and citations in Scopus. While nearly 7 million researchers have contributed to Scopus, Ioannidis et al. have selected researchers who have exceeded specified thresholds for various citation metrics. The database includes articles published between 1960 and 2019, and citations received between 1996 and 2019; thus citations are under-counted for researchers who have published before 1996. I have limited this analysis to researchers who published their first paper (in a journal indexed in Scopus) between 1990 and 2009, so that most citations would be correctly accounted for, and all researchers would have completed at least 10 years of their career. To facilitate interpretation of authorship patterns I restricted this analysis to researchers in the health sciences, corresponding to the fields of Clinical medicine, Biomedical research, Public health & Health services, and Psychology & Cognitive sciences [17].

### Variables

The dependent variables of the analyses were 2 measures of citation impact: the H-index [8], and the Hm-index [13]. The independent variables were the numbers of articles published as single author, first author, mid-list author, and last author. The database provided numbers of (a) single author articles (S), (b) single or first author articles (SF), (c) single, first or last author articles (SFL), and (d) total articles (T). I computed the numbers of first author articles as SF – S, mid-list author articles as T – SFL, and last author articles as SFL – SF.

### Analysis

To identify anomalies in the data, I examined the upper and lower ends of the frequency distributions of article counts. This led to the identification of authors who had mostly single author articles and non-academic affiliations; upon verification the majority were science journalists or editors, and their articles were incorrectly classified as original research. The 105 journalists/editors and 3 others affiliated with foundations or other non-academic entities were removed from the database.

The numbers of articles of each type and citation indices were described by their mean, standard deviation (SD), extreme values, and percentiles 25, 50, 75, and 95 (Table 1).

To examine to what extent the H and Hm indices capture the same information I obtained the Pearson correlation coefficient (r). The coefficient of determination r^2^ corresponds to the proportion of variance shared by the two indices.

**Fig. 1:**
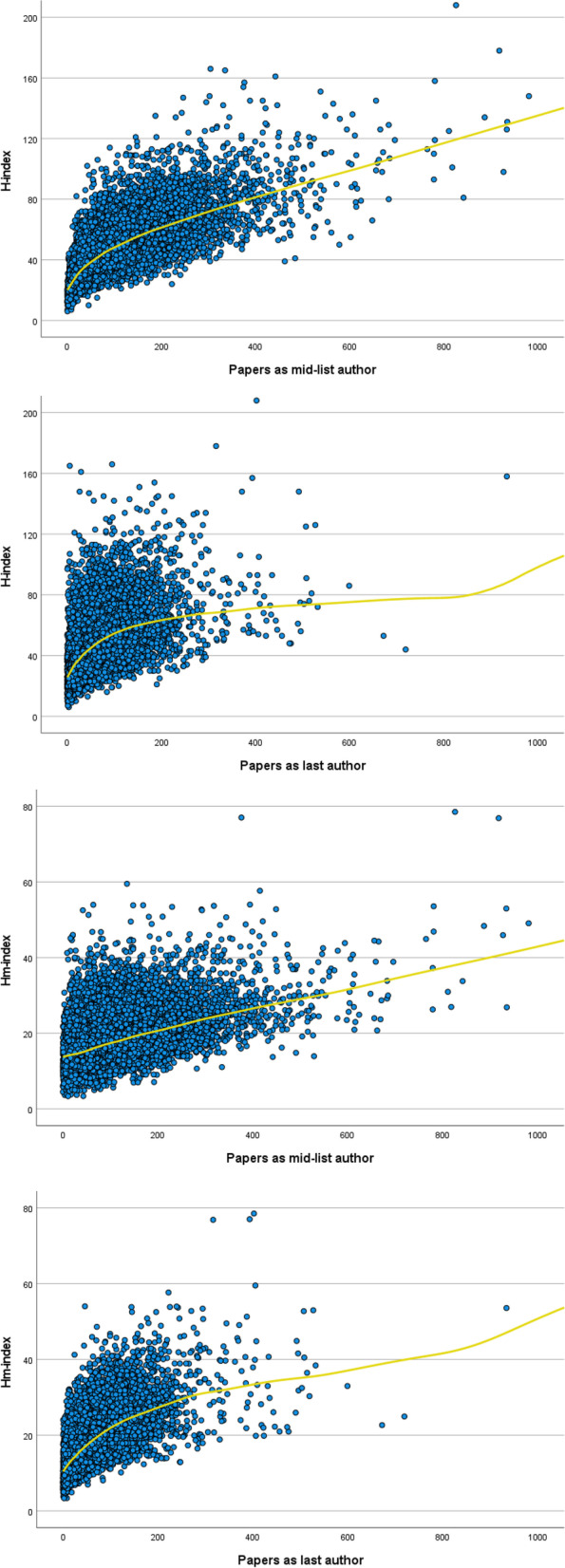
Scatter plots of the H-index (first and third panel) and Hm-index (second and fourth panel) as a function of the number of papers written as mid-list author (first and second panel) and as last author (third and fourth panel), among 18,231 leading researchers in the health sciences. Non-parametric (Lowess) regression lines are superimposed

**Fig. 2:**
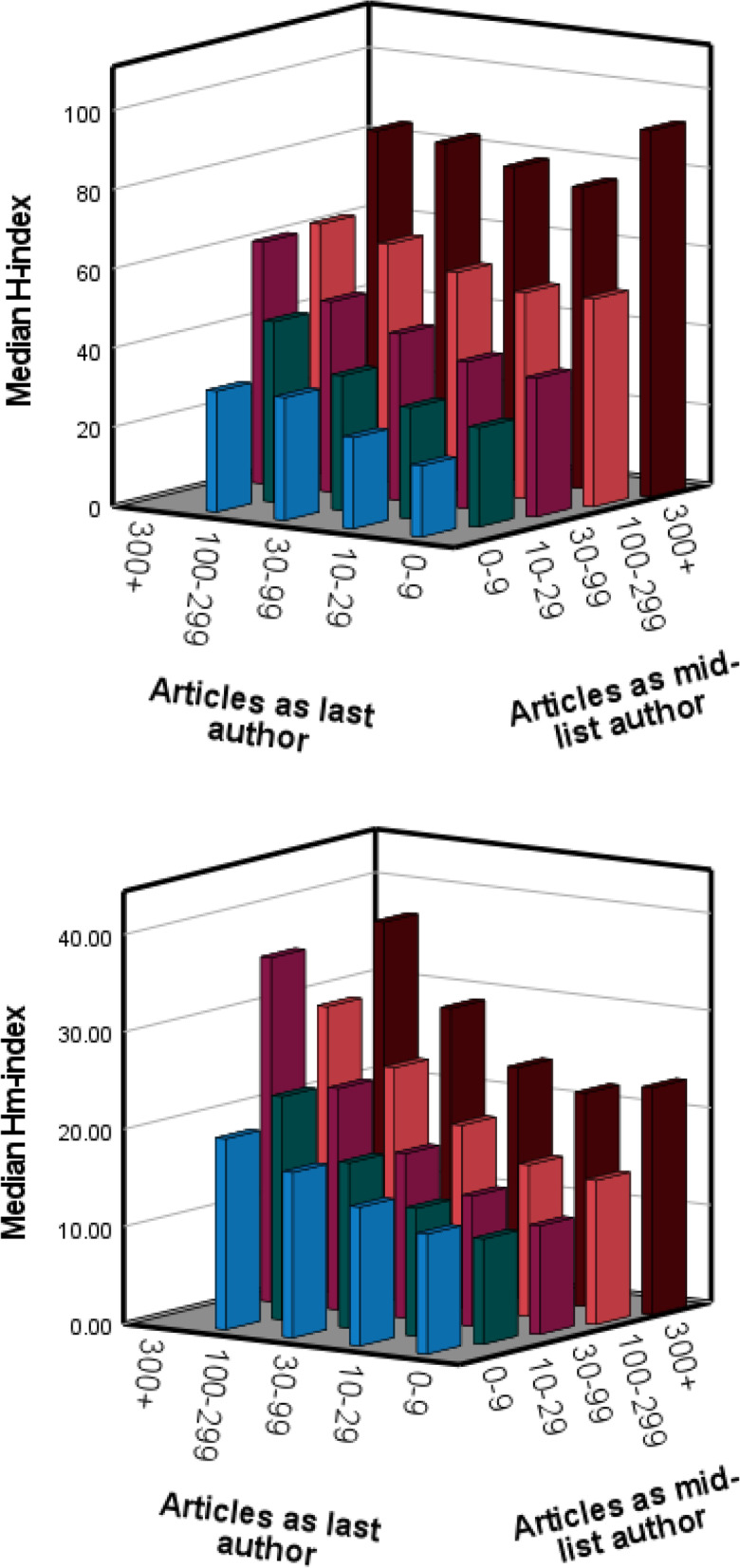
Median values of the H-index (upper panel) and Hm-index (lower panel) as a function of categories of numbers of articles written as last author and as mid-list author, among 18,231 leading researchers in the health sciences

Associations between the citation indices and numbers of articles as mid-list author and as last author were explored by means of scatter-plots (Fig. 1), and via bar-charts of median values of citation indices across categories of article numbers (Fig. 2).

Linear associations between article numbers and citation metrics were also examined by means of Pearson correlation coefficients (Table 2). The Pearson correlation coefficient corresponds to the expected change in one variable, in standard deviation units, for a one standard deviation increment of the other variable. To account for potential confounding, I obtained partial correlation coefficients, adjusting for career duration (reflected by the year of first publication) and for the numbers of other types of articles (this is equivalent to multiple linear regression).

To show the shape of the associations between numbers of articles and citation metrics without imposing a linearity assumption, I report observed means of the H-index and Hm-index for subsets of authors who have published 0–9, 10–29, 30–99, 100–299, and ≥ 300 articles of each type, as well as expected means after adjustment for other article types and for career duration (Table 3).

To examine the stability of the association patterns across scientific domains, linear associations were also analyzed separately for Clinical medicine, Biomedical research, Public health & Health services, and Psychology & Cognitive sciences (Table 4).

The analyses were performed using SPSS version 25.

## Results

### Sample characteristics

The complete database included 159,683 individual records; restriction to health-related scientific domains and to dates of first published paper between 1990 and 2009 yielded 18,339 records. After removal of 108 records of authors who were not affiliated with an academic institution and contributed mostly commentary (journalists and others), 18,231 individual records remained.

The majority of the researchers published their first paper between 1990 and 1995 (11,024, 60.5%), the rest did between 1996 and 2009 (7,207, 39.5%). The most common scientific field was Clinical medicine (12,858, 70.5%), followed by Biomedical research (3,270, 17.9%), Public health & Health services (1033, 5.7%), and Psychology & Cognitive sciences (1070, 5.9%).

### Distributions of articles and citation metrics

On average, each author published 188 articles (Table 1): 9 as single author, 30 as first author, 94 as mid-list author, and 55 as last author. The ranges were wide, as some authors contributed up to several thousand articles. The distributions of numbers of articles were skewed to high values, as illustrated by the spacing of quartiles (Table 1). Average values were 45.8 for the H-index, and 17.7 for the Hm-index (Table 1). These indices had nearly bell-shaped distributions, with a less marked positive skew. The Pearson correlation coefficient between the H-index and the Hm-index was 0.70, and the proportion of shared variance was 0.49.

**Table 1:** Distributions of the numbers of articles published and of citation metrics among 18,231 leading researchers in the health sciences

	Mean (SD)	Range	Proportion with 0 papers	Percentiles			
				25	50	75	95
Total articles	188 (131)	6–3050		103	156	236	429
Articles as single author	9 (18)	0–1672	2.93%	3	6	11	29
Articles as first author	30 (25)	0–796	0.04%	15	24	37	71
Articles as mid-list author	94 (83)	0–1153	0.10%	39	71	123	248
Articles as last author	55 (50)	0–1478	0.22%	25	43	70	140
H index	45.8 (17.0)	6 − 208		34	43	54	77
Hm index	17.7 (6.0)	3.4–78.5		13.7	16.8	20.5	28.9

### Exploratory analyses

The scatterplots of the H and Hm indices across the two main article counts (i.e., as mid-list and last author) show gradual associations with citation indices (Fig. 1). The non-parametric regression lines suggest a reasonable fit for linear models.

The median values of the H-index increased steeply across categories of mid-list authorships, but less so for last author contributions (Fig. 2, upper panel). The pattern was more balanced for the Hm-index, which increased regularly with both types of authorship (Fig. 2, lower panel).

### Linear associations between numbers of articles and citation metrics

The total number of articles published correlated with both the H-index (Pearson r 0.68) and the Hm-index (0.65). The H-index did not correlate with the number of single author articles, and only weakly with first author articles (Table 2). The correlations were strongest with mid-list author articles, and somewhat less strong with last author articles. After adjustment for career duration and other types of articles, the partial correlations with the H-index were weakly negative for single-author and first-author articles, but remained strong for mid-list authorships.

**Table 2:** Pearson correlation coefficients between numbers of articles published and citation metrics, unadjusted and adjusted for numbers of other articles and for career duration, among 18,231 leading researchers in the health sciences

	H-index		Hm-index	
	Unadjusted	Adjusted	Unadjusted	Adjusted
Single-author articles	-0.02	-0.06	0.18	0.04
First-author articles	0.17	-0.08	0.36	0.18
Mid-list-author articles	0.73	0.64	0.49	0.24
Last-author articles	0.48	0.21	0.63	0.46

In contrast, the Hm-index was correlated more evenly with article types (Table 2). The correlations were strongest with last-author articles, then with mid-list author articles, weaker yet with first author articles, and weakest, but not null, with single author articles. Adjustment reduced all these associations, but the pattern remained the same. Of note, due to the large sample size, all correlation coefficients in Table 2 were significantly different from 0.

### Associations for categorized article counts

Globally, these analyses (Table 3) confirm the analyses of linear trends, and demonstrate the gradual nature of the associations. The average H-index was stable across categories of single-author articles, and increased gradually across categories of the other types of articles. The strongest gradient was seen for mid-list authorships, as the average H was 22.5 for researchers who published 0–9 such papers, versus 84.9 for those who had ≥ 300 mid-list authorships. Adjustment attenuated these associations. The Hm-index increased gradually across all article categories, but most strongly for last-author articles: the average value of Hm was 11.9 for those with 0–9 last-author article, but 35.6 for those who had ≥ 300 last-author contributions. Adjustment attenuated the associations but maintained the same pattern.

**Table 3:** Average values of H-index and Hm-index across categories of numbers of articles written as single author, first author, mid-list author, and last author, observed (unadjusted) and adjusted for other article types and career duration, among 18,231 leading researchers in the health sciences

		H-index		Hm-index	
Single-author articles	Percent	Observed	Adjusted	Observed	Adjusted
0–9	69.7%	45.9	47.3	16.8	21.8
10–29	25.6%	45.8	46.8	19.4	23.3
30–99	4.4%	46.3	46.7	22.5	25.2
100–299	0.3%	42.4	46.1	23.9	26.7
≥ 300	0.0% (N = 3)	45.3	34.2	22.1	18.9
First-author articles					
0–9	9.4%	41.9	48.9	14.5	20.4
10–29	53.5%	44.3	47.5	16.6	21.4
30–99	35.1%	48.8	46.5	19.9	22.6
100–299	1.9%	56.0	45.6	25.4	24.7
≥ 300	0.0% (N = 6)	67.0	32.5	37.2	26.7
Mid-list-author articles					
0–9	2.4%	22.5	23.3	14.1	21.8
10–29	14.0%	30.8	29.6	14.9	21.3
30–99	49.2%	41.6	38.7	16.5	21.5
100–299	31.6%	57.5	52.6	20.2	23.1
≥ 300	2.7%	84.9	77.0	28.4	28.2
Last-author articles					
0–9	5.9%	31.1	35.2	11.9	16.2
10–29	26.2%	37.7	37.9	14.1	18.1
30–99	55.8%	47.6	42.5	18.3	21.5
100–299	11.7%	61.8	48.7	25.4	26.5
≥ 300	0.4%	79.6	56.8	35.6	33.6

### Linear associations across scientific domains

The pattern of linear associations between numbers of articles and the H-index and Hm-index was similar for the majority domain, Clinical medicine, as for the pooled analysis (Table 4). The key finding, i.e., that the correlation coefficients were strongest for mid-list author papers and the H-index on the one hand, and for last-author papers and the Hm-index on the other hand, held across all 4 domains. Beyond this, the strength of the observed associations varied moderately across scientific domains.

**Table 4:** Pearson correlation coefficients between numbers of articles published and citation metrics, unadjusted and adjusted for numbers of other articles and for career duration, stratified by scientific field, among 18,231 leading researchers in the health sciences

	H-index		Hm-index	
	Unadjusted	Adjusted	Unadjusted	Adjusted
Clinical medicine (*N* = 12,858, 70.5%)				
Single-author articles	0.00	-0.05	0.17	0.04
First-author articles	0.17	-0.06	0.35	0.20
Mid-list-author articles	0.74	0.65	0.55	0.32
Last-author articles	0.47	0.18	0.63	0.44
Biomedical research (*N* = 3270, 17.9%)				
Single-author articles	-0.04	-0.02	0.26	0.25
First-author articles	0.10	-0.20	0.32	0.11
Mid-list-author articles	0.70	0.66	0.41	0.21
Last-author articles	0.45	0.28	0.71	0.63
Public health & Health services (*N* = 1033, 5.7%)				
Single-author articles	-0.17	-0.14	0.16	0.26
First-author articles	0.41	0.25	0.51	0.30
Mid-list-author articles	0.78	0.61	0.61	0.35
Last-author articles	0.61	0.17	0.65	0.37
Psychology & Cognitive sciences (*N* = 1070, 5.9%)				
Single-author articles	0.00	-0.17	0.28	0.14
First-author articles	0.37	0.21	0.50	0.32
Mid-list-author articles	0.67	0.46	0.48	0.15
Last-author articles	0.62	0.39	0.67	0.52

## Discussion

This analysis showed that among highly cited scientific authors, the H-index and the Hm-index were both similarly correlated with the total number of authored articles. However, the two indices were not associated with the same authorship patterns, as reflected by the numbers of articles published as single, first, mid-list, and last author. Notably, the H-index was strongly associated with the number of articles written as mid-list author, but the Hm-index was more strongly dependent on the number of papers signed as last author. This key result held across the four main scientific domains.

The prediction that H would correlate more closely than Hm with the total number of articles written was not really borne out by the data (while 0.68 is greater than 0.65, the order of magnitude is similar). Thus both indices reflected similarly the total number of published articles. The chief contrast between the indices resided in their responsiveness to different types of author positions, which in turn reflect the roles assumed by scientists in the course of research.

A striking finding was the strong positive impact of mid-list author contributions on the H-index, and the seemingly detrimental effect (in adjusted models) of single-author or first-author papers. In interpreting these results, and particularly the negative associations, one should recall that the regression models are not causal models but post-hoc descriptions, and that adjusted effects represent associations subject to other effects being held constant. But in real life all things are not held constant; a researcher who invests time and energy into leading her own project (to plan, conduct, analyze, write up and publish as first author) has less availability for collaborations. This analysis shows that researchers who got repeatedly involved in projects led by others (and thus appeared as mid-list coauthors) reached higher H-index values than those who invested in first- or last-author roles. This may be seen as a weakness of the H-index, since mid-list authorships do not reflect a researcher’s most important personal contributions to science. Furthermore, mid-list authorships are also most vulnerable to manipulation, e.g. via gift authorship. These concerns suggest that H-index values should be used with caution when assessing researchers’ careers.

Why does a paper in which the researcher contributed as a mid-list author accrue *more* H-index points than a paper the researcher wrote as a first or last author? One possibility is that collaborative projects produce consistently higher quality science that will get cited more. Indeed, ambitious scientific endeavors – multi-center clinical trials, meta-analyses of genome-wide association studies, consensus statements, pooled cohorts or registries – often require vast collaborative networks, and many such multi-authored papers become citation classics [18]. Of note, the positive relationship between the number of authors and the number of citations at the article level has been observed repeatedly [19–21]. Another explanation may be the compounding effect of collective self-citation. Self-citations may serve other purposes than signaling scientific utility; e.g., they may provide linkage with previous work of the research team and avoid duplication [22]. Such self-referential usage would naturally increase with the number of coauthors, and thus inflate citation metrics.

In contrast, the Hm-index was sensitive to all types of author contributions, and most strongly to the number of last-author papers. The greater importance of first and last author contributions for the Hm-index reflects the greater relative weight of articles with short author lists: e.g., first and last positions represent 50% of author positions for a paper written by 4 coauthors, but only 10% if there are 20 coauthors. These results confirm that the choice of performance metrics have a potential for influencing the practice of science. Importantly, deliberate optimization of one’s indicators by individual researchers is not required, as performance indicators may act through passive selective pressure [5]. Consider two researchers: the one whose performance metric X is higher will be promoted, and the other will leave the field; then academic success will be associated with X, seemingly demonstrating the validity of X. It seems therefore that H should be preferred if policymakers wished to promote multiple collaborations, and Hm if policymakers wished to promote a more balanced portfolio, with focus on research leadership.

### Limitations

A limitation of this study is that as it uses historical data, the described associations may not reflect current practice or future trends. Secondly, for researchers who started publishing before 1996, the citation metrics are incomplete, since in Scopus citations were counted between 1996 and 2019; however it seems unlikely that this selection would have influenced differently the associations between authorship and the two indices. Thirdly, the database contains only 2–3% of scientific authors [17], those at the upper end of the citation spectrum; whether the associations between articles and citation metrics are similar among less cited researchers is unclear. Furthermore, in absence of unique individual identifiers, homonymy may have led to incorrect identification or aggregation of some researchers. Other limitations of the dataset include incomplete coverage of conference papers or of the grey literature, and incomplete recognition of joint first or last authorship.

Other limitations stem from a lack of information about key variables. Authors’ roles as researchers were unknown, as only the distribution of numbers of articles by author position was available. Misconduct, such as gift authorship or unrecognized contributions, could not be addressed. Finally, the validity of citations as indicators of research quality is debatable; indeed citations are influenced by factors other than scientific value [23–26], and are not necessarily associated with methodologic rigor or originality of the underlying science. As both H and Hm are based on citations, neither index rises above these limitations.

Other citation-based indicators than H and Hm have been proposed, and others yet might be developed. E.g., if first or last author positions were of particular interest, citation indicators might be obtained separately for these roles [27]; indeed, first and last authors do, on average, contribute more than others to a given research project [28, 29].

## Conclusion

This study shows that citation metrics do not assign value to authorship roles in the same way: the H-index is highest among researchers who contribute predominantly as mid-list authors, whereas the Hm-index is highest among those who contribute frequently as last authors. Because performance metrics act as incentives, choosing the appropriate metric may influence the way science is done. Reliance on the H-index, which is currently common in academic settings, risks encouraging limited contributions to multiple research projects, and discouraging in-depth engagement in one’s own research, since the payoff is greater for the former than for latter. Reliance on the Hm-index, which is currently uncommon to our knowledge, encourages a more balanced scientific port-folio; while mid-list contributions also increase the Hm-index, their impact is less strong than that of last author contributions.

Finally, given the limitations of citation-based metrics, a reasonable recommendation would be to broaden the scope of researchers’ evaluations, and include for instance assessments of societal impact, and in-depth analysis of key research projects.

### Supplementary Information

**Additional file 1.**

## Data Availability

The study used publicly available data (Ioannidis JPA, Boyack KW, Baas J. Updated science-wide author databases of standardized citation indicators. PloS Biology 2020;18:e3000918). The data selected for this analysis are available as a supplement.

## Abbreviations

SD: Standard deviation (defined on line 119)
H-index: H stands for Hirsch, the author (defined on line 53)
Hm-index: Hm is not an abbreviation; m signifies multiple authorship (line 57)
